# The growing field of liquid biopsy and its Snowball effect on reshaping cancer management

**DOI:** 10.1016/j.jlb.2025.100293

**Published:** 2025-03-27

**Authors:** Roberto Borea, Carolina Reduzzi

**Affiliations:** a Department of Public Health, University Federico II of Naples, Naples, Italy; b Department of Internal Medicine and Medical Sciences (DiMI), School of Medicine, University of Genova, Genova, Italy; c Department of Medicine, Weill Cornell Medicine, Englander Institute for Precision Medicine, New York Presbyterian Hospital, New York, NY, 10021, USA

**Keywords:** Circulating tumor DNA/ctDNA, Circulating tumor cells/CTCs, MicroRNA/miRNA, Liquid biopsy, Solid tumors

## Abstract

Liquid biopsy (LB) has emerged as a transformative tool in oncology, providing a minimally invasive approach for tumor detection, molecular characterization, and real-time treatment monitoring. By analyzing circulating tumor DNA (ctDNA), circulating tumor cells (CTCs), extracellular vesicles (EVs), and microRNA (miRNA), LB enables comprehensive tumor profiling without the need for traditional tissue biopsies. Over the past decade, research in this field has expanded exponentially, leading to the integration of LB into clinical practice for specific cancer types, including lung and breast cancer. In 2024, the Journal of Liquid Biopsy (JLB) published innovative studies exploring the latest advancements in LB technologies, biomarkers, and their applications for cancer detection, minimal residual disease (MRD) monitoring, and therapy response assessment. This review synthesizes recent findings on the role of LB in cancer treatment and monitoring across different biomarkers, with a particular focus on newly published studies and their context within translational research. Additionally, it highlights emerging techniques such as fragmentomics, artificial intelligence, and multiomics, paving the way for more precise, personalized treatment decisions. Despite these advancements, challenges remain in standardizing methodologies, optimizing clinical validation, and integrating LB into routine oncological workflows. This mini-review highlights the evolving landscape of LB research and its potential to revolutionize cancer diagnosis, treatment monitoring, and therapeutic decision-making, ushering in a new era of precision oncology.

## 1 Introduction

Liquid biopsy (LB) has revolutionized the management of solid tumors, enabling applications in early cancer detection, diagnosis, treatment monitoring, predictive biomarker for therapy selection, and prognostic assessment [1,2]. While many of these applications remain under investigation, LB has already been incorporated into clinical practice for specific tumor subtypes. Examples include its role in the molecular characterization of lung cancer and the detection of estrogen receptor alpha (*ESR1*) mutations in patients with hormone receptor (HR) positive/human epidermal growth factor receptor 2 (HER2) -negative metastatic breast cancer [3,4].

**Fig. 1:**
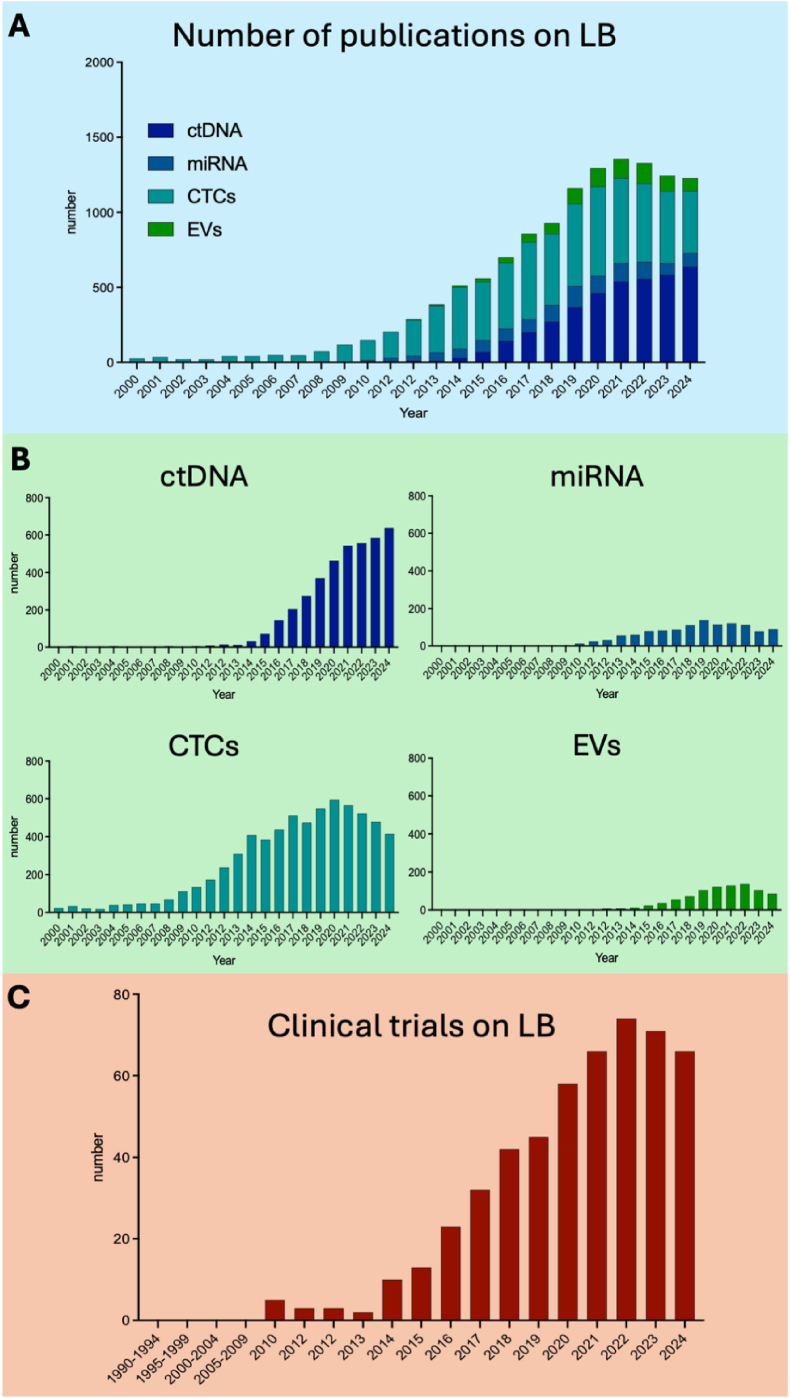
Evolution of liquid biopsy field in the last two decades. A) Summary of LB original articles on different topics B) Number of original articles on specific biomarkers C) Number of clinical trials using liquid biopsy opened by year. Abbreviations: LB, Liquid Biopsy; ctDNA, circulating tumor DNA; miRNAs, microRNA; CTCs, circulating tumor cells; EVs, extracellular vesicles.

The rapid expansion of the LB field is reflected in the exponential increase in scientific publications on this topic. In 2015, fewer than 1000 studies were published in this field, whereas by 2024, this number has surpassed 3,000, and it continues to rise (Fig. 1A) (search string is available in the supplementary file). Furthermore, besides the four primary areas of LB research: circulating tumor DNA (ctDNA) [5], microRNAs (miRNAs) [6], circulating tumor cells (CTCs) [7], and extracellular vesicles (EVs) [8], new biomarkers such as platelets [9], and proteins [10] have been investigated in the last few years. Notably, the growth rate in each of these subfields has remained relatively consistent over time (Fig. 1B). This wave in publications correlates with an increasing number of clinical studies integrating LB into their protocols, aiming to enhance tumor profiling and identify novel circulating biomarkers (Fig. 1C). Such prospective trials are essential for the implementation of LB in clinical practice. New (neo)adjuvant studies have been designed to allow de-escalation or escalation treatment based on LB biomarkers [11,12]. In parallel with growing scientific evidence supporting the clinical utility of LB, efforts have been made to develop standardized guidelines for its implementation in daily clinical practice. One such initiative is the Liquid Biopsy Response Evaluation Criteria in Solid Tumors (LB-RECIST), designed to establish response criteria for liquid biopsy-based monitoring [13]. However, despite significant advancements, several challenges remain before LB can be fully integrated into routine clinical practice. To further support advancements in this field and consolidate the growing body of research, the International Society of Liquid Biopsy (ISLB) recently established the Journal of Liquid Biopsy (JLB), a dedicated scientific journal focused exclusively on LB research. In this mini-review, we analyze articles published in the JLB in 2024, to highlight the key analytes, methodologies, and emerging technologies that are shaping the future of LB in oncology. By examining the latest research, we aim to provide insights into the evolving landscape of LB and its potential to revolutionize cancer diagnosis, treatment monitoring, and personalized therapy.

## 2 2024 findings reported in JLB

### 2.1 ctDNA

ctDNA, the most studied circulating biomarker currently utilized in routine clinical practice in different cancer types, represents the fraction of cell-free DNA (cfDNA) that is derived from tumor tissue [14]. Given that ctDNA levels vary according to tumor stage and type, the application of ctDNA-based LB depends on clinical use. In the context of early cancer detection and screening, as well as during the assessment of minimal residual disease (MRD), where tumor burden is minimal, a highly sensitive assay is mandatory [15]. Furthermore, ctDNA MRD can be exploited in two main strategies: tumor-informed and tumor-agnostic. The tumor-informed approach involves monitoring known patient-specific primary tumor variants, while the tumor-agnostic one evaluates tumor signals in cfDNA (comprising mutations, copy number changes and methylation patterns) independently from the primary tumor tissue [16,17]. Conversely, in advanced-stage malignancies, where tumor burden is higher, next-generation sequencing (NGS)-based comprehensive genomic profiling (CGP) or targeted genomic profiling are more appropriate for identifying actionable genomic alterations to guide therapeutic decision-making [18]. Additionally, multiple factors may affect both the analytical and clinical validity of ctDNA assays [19]. Considering this, standardized practices and guidelines outline pre-analytical requirements for ctDNA analysis, including rigorous quality control measures for blood collection and DNA extraction [19]. Nevertheless, every year new technologies are becoming available and the recommendations must take into consideration new possible approaches to ctDNA analysis. In the context of metastatic disease, De Giglio et al. investigated the prevalence of Kirsten rat sarcoma viral oncogene (*KRAS*) exon 2 p.G12C mutations in a consecutive cohort of 24 patients with non-small cell lung cancer (NSCLC). At baseline (T0), ctDNA was detectable in 17 out of 24 patients (70.8 %). Specific groups of patients, such as subjects with baseline liver metastases (4/24, 16.7 %) exhibited a significantly higher mean ctDNA concentration compared to those without liver metastases (*p* = 0.01), with a median allele frequency (AF) at T0 of 1.2 % (interquartile range [IQR]: 0–9.9). Furthermore, the presence of three or more metastatic sites was associated with a significantly higher mean T0 AF (17.4 %) compared to patients with one or two metastatic sites (1.2 %) (*p* = 0.002). Patients were stratified into high-AF and low-AF groups based on the median T0 AF (1.2 %). The median overall survival (OS) was 7.5 months (95 % confidence interval [CI]: 1.91–not reached) in the high-AF group and 11.3 months (95 % CI: 6.6–not reached) in the low-AF group (*p* = 0.38) [20]. This study aligns with ongoing advancements in lung cancer research, particularly by demonstrating that digital droplet PCR (ddPCR) for the detection of a single genetic alteration can serve as a viable alternative to NGS [21,22]. Additionally, these findings reinforce the prognostic relevance of plasma AF reduction during systemic treatment, which has been associated with improved tumor response and prolonged survival outcomes [23]. As previously discussed, NGS and ddPCR remain the two primary methodologies for analyzing genetic alterations in specific genes, with each technique possessing distinct characteristics that enable different types of analyses. In a study by Scimone et al., the concordance of NGS and ddPCR was assessed in a retrospective cohort of 50 patients with NSCLC who had previously undergone NGS panel testing for *KRAS/*epidermal growth factor receptor (*EGFR*) alterations, showing the presence of mutations in half of the cohort and a wild-type tumor in the remaining half. ddPCR successfully identified 24 out of 25 mutated cases and 21 out of 24 wild-type cases. When applying a mutant allele frequency (MAF) cut-off of ≥0.2 % and a threshold of 100 positive partitions in the wild-type channel, the study reported a technical sensitivity of 96.0 % (24/25), specificity of 88.0 % (22/25), and an overall concordance rate of 92.0 % (46/50) [24]. Similarly to previous findings, this study supports the use of dPCR as a reliable molecular diagnostic tool for routine clinical specimens [20,21].

In the context of molecular alteration analysis, not all genetic changes exhibit similar characteristics. Alterations in the mesenchymal epithelial transition (*MET*) gene, encoding a tyrosine kinase receptor, are frequently observed in NSCLC, with *MET* exon 14 skipping mutations leading to increased receptor activity [25]. Despite the widespread use of NGS for ctDNA analysis in advanced NSCLC, its effectiveness in detecting gene rearrangements remains limited due to technical challenges, particularly in LB samples with low ctDNA content. While circulating tumor RNA (ctRNA) could improve detection, its low quantity and quality hinder routine clinical use. However, gene rearrangement prevalence in tissue and LB was comparable when tumor fraction (TF) was ≥1 % [26]. To assess NGS-based detection, two Italian centers analyzed 58 patients with known oncogenic fusions or *MET* exon 14 mutations. The overall Positive Percent Agreement (PPA) was 37 % (21/57), with the highest performance for rearranged during transfection (*RET*) fusions (80 %), intermediate for METexon14 skipping mutations (40 %) and anaplastic lymphoma kinase (*ALK*)-rearranged (36 %), and the lowest for ROS proto-oncogene 1, receptor tyrosine kinase (*ROS1*) rearrangements (18 %) [27]. This study confirms that ctDNA analysis is a valuable tool for detecting *MET* exon 14 mutations and gene fusions in advanced NSCLC [[27], [28], [29]]. However, detection rates vary by mutation type and clinical factors. Similarly to NSCLC, in advanced breast cancer (BC), LB is growing as a tool for treatment decision making. In advanced estrogen receptor–positive (ER+) BC, *ESR1* alteration is acquired during endocrine therapy and is rarely present at diagnosis. *ESR1* mutation detection has become clinically relevant, prompting an American Society of Clinical Oncology (ASCO) Guideline Rapid Recommendation Update [30]. ctDNA enables reliable, real-time monitoring, leading to FDA approval of the Guardant360 CDx test for *ESR1*-mutated ER + metastatic BC patients with progressive disease (PD) on disease after endocrine therapy [31,32]. Smilkou et al. evaluated a novel 12plex *ESR1*- AKT Serine/Threonine Kinase 1 (*AKT*) 6-color Crystal Digital PCR® assay for detecting *ESR1* mutations and *AKT1* E17K in plasma cfDNA collected from patients with ER + BC. Among 35 samples, the assay detected both *AKT1* p.E17K and *ESR1* p.D538G in 5/35 (14.3 %) cases, while *ESR1* p.D538G alone was found in 4/35 (11.4 %) using the *ESR1* NaME-PrO-assisted ARMS (NAPA) assay. A direct comparison showed high concordance (97.1 %, κ = 0.871, *p* < 0.001) for *ESR1* p.D538G detection. Considering these results, the Stilla 12plex *ESR1-AKT1* 6-color Crystal Digital PCR® assay demonstrated to be a highly sensitive, multiplex method for LB, offering a robust approach for detecting *ESR1* and *AKT1* mutations in ER + BC [33]. This is particularly relevant, as ddPCR plays a crucial role in the assessment of molecular targets in BC, as previously reported in several studies [34,35]. Advancing these techniques can provide significant benefits by enabling more precise detection of actionable alterations, ultimately supporting optimized treatment strategies for patients with advanced BC. Similarly to its role in advanced disease, LB is being investigated in early-stage BC to assess relapse risk and potential treatment benefits. In triple-negative breast cancer (TNBC), the advent of immunotherapy has transformed both neoadjuvant and adjuvant treatment strategies [36]. While several trials have evaluated the addition of immune checkpoint inhibitors (ICIs) in these settings, Keynote-522 remains the only study to demonstrate a positive outcome [37,38]. Within this context, Ahmed et al. assessed the role of LB in a clinical trial (NCT02489448) investigating Durvalumab plus chemotherapy in stage I–III TNBC [39]. Using the PredicineBEACON ultra-deep tumor-informed sequencing assay and the PredicineEPIC cfDNA methylation assay, they analyzed 44 TNBC patient samples. A significant positive correlation was observed between residual cancer burden (RCB) scores and post-neoadjuvant chemotherapy (NAC) TF (*r* = 0.45, *p* = 0.004). Median TF was lower in patients achieving pathologic complete response (pCR, RCB0) compared to those with residual disease (0.06 % vs. 0.3 %, *p* = 0.02) [40]. Moreover, using a TF positivity threshold of ≥0.05 %, PredicineBEACON achieved 58 % sensitivity and 83 % specificity for detecting residual disease. Additionally, TF was significantly higher in patients who experienced recurrence (0.17 % vs. 0.05 %, *p* = 0.029). Further, post-NAC methylation signals were reduced compared to pre-treatment levels, with lower post-treatment methylation observed in pCR cases versus residual disease ones [40]. This study, similarly to previous reports, highlights how post-NAC plasma tumor fraction and changes in tumor-derived methylation signal are predictive of residual disease burden and recurrence risk in TNBC patients [41]. These findings highlight the potential of ctDNA-based LB as a valuable tool for early treatment response assessment and disease monitoring. Highly sensitive and specific MRD detection assays have the potential to revolutionize treatment in TNBC and other early-cancer tumors. These assays could facilitate objective MRD-based therapy de-escalation or escalation strategies. This approach is currently being prospectively tested in the I-SPY2.2 clinical trial (NCT01042379) [42]. Additionally, ctDNA surveillance during post-treatment follow-up could identify patients at risk of recurrence who may still have microscopic disease. Different ongoing prospective trials in the United States, such as DARE and LEADER (NCT03285412) for estrogen receptor-positive breast cancer, and ASPIRA (NCT04434040) and PERSEVERE (NCT04849364) for TNBC, are testing the clinical utility of ctDNA monitoring during follow-up. In cfDNA analysis, fragmentomics is also emerging as a potential new approach. Besides various techniques for LB analysis, such as dPCR and NGS [43,44], the concordance between mutations identified in lung tumor tissue and plasma using targeted sequencing is approximately 75 %, with even lower detection rates for oncogenic gene fusions [45]. To enhance ctDNA detection relative to non-tumor cfDNA, recent studies have identified distinct biological characteristics of ctDNA, including its shorter fragment length [46,47]. A study by Trier Maansson et al. investigated the impact of in vitro size selection on ctDNA analysis in patients with lung cancer. Plasma samples were analyzed with and without size selection using targeted sequencing to evaluate its effect on MAF and genome-wide copy number alterations (CNA). Results showed that tumor-derived ctDNA exhibited a distinct fragment length profile compared to cfDNA fragments containing clonal hematopoiesis (CH) or germline mutations, which often lead to false-positive findings. Size selection increased the median MAF of tumor mutations by 1.36-fold (IQR: 0.63–2.48), whereas CH/germline mutations showed minimal enrichment (median 0.95-fold, IQR: 0.62–1.05) [48]. Moreover, key oncogenic drivers, such as *KRAS* and *EGFR*, demonstrated greater MAF enrichment following size selection. Additionally, the number of plasma aneuploidy-positive samples increased from 8/35 to 20/35 [48]. This study, the largest of its kind analyzing 35 patients with 109 mutations, highlights the potential of fragmentomics and in vitro size selection for improving ctDNA detection and analysis. This study shed light on fragmentomics, which is emerging as a new tool in ctDNA analysis, providing valuable prognostic insights beyond standard mutation-based detection. Its integration into targeted ctDNA panels offers additional biological information without requiring extra sequencing costs or additional sample input. Recent studies have demonstrated that fragmentomics can accurately define tumor vs normal, as well as tumor types, even at low ctDNA fractions [49,50]. This is particularly relevant in cancers with minimal ctDNA shedding, where conventional mutation-based approaches may lack sensitivity. Furthermore, fragmentomics-based metrics have shown potential prognostic value, correlating with tumor burden, treatment response, and disease progression across various malignancies [49]. One of the key advantages of fragmentomics is its applicability to existing commercial targeted sequencing assays, as it does not require germline sequencing for ctDNA fraction estimation. This enables a comprehensive assessment of tumor-derived alterations while maximizing the clinical utility of a single plasma sample. While most LB studies rely on peripheral blood, increasing attention is being given to alternative biofluids for identifying circulating biomarkers. Among these, saliva has shown promise for head and neck cancers [51], urine for genitourinary tumors [52,53], and cerebrospinal fluid (CSF) for gliomas [54,55]. However, due to the invasive nature and higher complication risks of CSF collection, Karacam et al. focused on analyzing cfDNA and extracellular vesicles (EVs) in the blood of patients with glioma. In their study, serum cfDNA and EVs levels were analyzed in 17 patients undergoing surgical resection. Consistent with previous research, mean serum cfDNA levels were higher in glioma patients compared to healthy controls before surgery, though the difference was not statistically significant [56,57]. Notably, cfDNA levels exhibited a decreasing trend following surgery, with a significant reduction observed at three months post-resection, particularly evident in patients without relapse (*p* = 0.007) [57]. Similarly, EV levels were significantly elevated in glioma patients compared to controls at diagnosis (*p* = 0.002), supporting previous findings by Osti et al. [57,58] These findings highlight the potential role of cfDNA as a biomarker for glioblastoma, particularly in monitoring post-surgical disease dynamics and identifying patients at higher risk of recurrence.

### 2.2 CTCs

Tumor spread and metastasis is a complex biological process involving local invasion, systemic dissemination, extravasation, and colonization in distant organs. Circulating Tumor Cells (CTCs) play a critical role in this process, as they detach from the primary tumor and enter circulation via the lymphatic system or bloodstream [7]. From a clinical perspective, CTCs provide a non-invasive tool for real-time monitoring of tumor heterogeneity, cellular senescence, MRD detection, and metastatic potential [[59], [60], [61]]. Elevated CTC counts have been correlated with reduced OS and Progression-Free Survival (PFS), reinforcing their prognostic significance in monitoring disease progression and therapeutic response [7]. A systematic review by Bahmaie et al. provided an extensive overview of the clinical applications of CTCs, highlighting their diagnostic, prognostic, and therapeutic potential, in the context of immune-oncology. The study emphasized that CTCs counts are strongly influenced by tumor type and stage, and their dissemination in circulation appears to be modulated by specific immune cell interactions. Additionally, the optimization of CTC isolation techniques remains a crucial aspect in enhancing their detection and clinical utility [62]. A novel concept, termed Cellular Residual Disease (CRD), is gaining attention in the context of early MRD detection [60]. A recent study introduced a combinational index called P-score to improve disease monitoring in patients with lung cancer. This analysis included 13 patients with 54 blood samples, assessing both CTCs and circulating tumor microemboli (CTM). The P-score demonstrated superior accuracy compared to individual CTC/CTM measurements by reducing false positives. Statistically significant differences were observed in CTC and CTM distribution in PD (*p* = 0.0018) and stable disease (SD) (*p* = 0.000048) samples. The P-score-based longitudinal monitoring aligned closely with clinical outcomes, exhibiting greater sensitivity than single biomarkers. These findings suggest that incorporating CRD alongside MRD could enhance early cancer detection and treatment monitoring [63]. This study underscores the expanding role of CTCs beyond traditional MRD assessment, offering new insights into their prognostic relevance and potential integration into personalized cancer management.

### 2.3 RNA analysis

While CTCs and ctDNA have been the primary focus of LB research, increasing attention is being directed toward the analysis of RNA as a possible biomarkers. Circulating cell free RNA (cfRNA) consists of fragmented RNA molecules released into the bloodstream, providing insights into both apoptotic/necrotic tumor cells and actively secreting tumor cells through exosome-mediated signaling [64]. However, a major limitation of cfRNA is its inherent instability, as it is highly susceptible to degradation by ribonucleases, making its isolation and analysis challenging [65]. Despite these challenges, several studies have employed messenger RNA (mRNA) expression panels to assess prognosis, metastatic potential, and recurrence risk across various cancer types [66,67]. Due to their greater stability and abundance, non-coding RNAs (ncRNAs), particularly small RNAs, are emerging as promising diagnostic and prognostic biomarkers [68]. The detection of ncRNAs in different biofluids further supports their potential role as stable LB biomarkers [69]. In this context, Walker et al. investigated the prognostic value of PD-L1 expression in cfRNA in a retrospectively enrolled cohort of patients with advanced NSCLC treated with ICIs. Their findings demonstrated that PD-L1 expression in cfRNA was comparable to PD-L1 protein expression in tumor tissue, with a median OS of 15 months and a 3-year OS rate of 30 % (HR: 0.97; 95 % CI: 0.44–2.10; p = 0.93) in PD-L1 positive patients [70]. Moreover, patients with positive plasma cfRNA PD-L1 expression exhibited a numerically longer median OS and higher 3-year OS compared to those lacking PD-L1 expression (median OS: 15 months vs. 8 months; 3-year OS: 30 % vs. 15 %; HR: 0.57; 95 % CI: 0.26–1.20; *p* = 0.11) [70]. This study reinforces the role of LB in monitoring immunotherapy response, complementing previous studies that have utilized ctDNA [71] and EVs [72] as predictive biomarkers. These findings highlight the potential clinical utility of cfRNA analysis in guiding immunotherapy strategies and improving patient outcomes. Beyond its established role in evaluating response to immunotherapy, LB also holds promise for assessing the efficacy of chemotherapy. Chemotherapy remains the standard of care for several malignancies, particularly those lacking actionable molecular targets or where the addition of ICIs has not demonstrated a clear clinical benefit. Pancreatic ductal adenocarcinoma (PDAC) exemplifies a tumor type in which ICIs have no current indication, and chemotherapy remains the cornerstone of treatment [73]. Given the potential utility of mRNA analysis in pancreatic cancer [[74], [75], [76]], a recent study investigated the predictive value of circulating plasma miRNAs for selecting the optimal chemotherapy regimen—either FOLFIRINOX or gemcitabine-nab-paclitaxel—in patients with advanced PDAC. NGS was conducted on pre-treatment plasma samples to identify candidate biomarkers, followed by validation in a cohort of 40 patients using reverse transcriptase quantitative polymerase chain reaction (RT-qPCR). Cox regression analysis was employed to determine the predictive capacity of specific plasma miRNAs for chemotherapy response. The study revealed that high plasma miR-379 expression was significantly predictive of treatment response (P_interaction_ = 0.0004) [77]. Among patients with low plasma miR-379 levels, those treated with FOLFIRINOX exhibited superior OS compared to those receiving gemcitabine-nab-paclitaxel (HR: 0.32; 95 % CI: 0.08–0.98; P = 0.046) [77]. Notably, other miRNAs, including miR-127, miR-155, and miR-200, did not exhibit predictive value for chemotherapy response (P_interaction_ = 0.12, 0.83, and 0.12, respectively). These findings suggest that plasma miR-379 may serve as a clinically relevant predictive biomarker to guide chemotherapy selection in advanced PDAC, potentially optimizing treatment outcomes. However, before its integration into routine clinical practice, further validation in larger, independent cohorts is necessary to confirm its predictive utility and establish its role in personalized treatment strategies for pancreatic cancer. As previously reported, the transcriptomic analysis of cfRNA enables the detection of mutations, gene fusions, and chromosomal translocations [78]. Additionally, cfRNA profiling offers valuable insights into the immune response, including cytokine and chemokine levels produced by both the tumor and the host [79]. Albitar et al. conducted a large-scale transcriptomic analysis of cfRNA using hybrid capture NGS in 1009 peripheral blood samples collected among patients affected by different cancer types. Their study demonstrated that by applying an artificial intelligence (AI) model, they could effectively differentiate between normal controls (N = 368) and patients with solid tumors (N = 404) with an area under the curve (AUC) of 0.820 (95 % CI: 0.760–0.879) [80]. Moreover, specific cancer subtypes, including lung, breast, colorectal, and myelodysplastic syndromes, were identifiable through this approach. These findings highlight the transformative potential of liquid transcriptomics when combined with AI and multi-omics profiling, shifting LB from a molecular tool for detecting actionable mutations and MRD to a comprehensive diagnostic platform [[81], [82], [83]].

### 2.4 Other biomarkers

Autoantibody production against tumor-associated antigens can arise due to various mechanisms, including oncogenic mutations, neo-antigen emergence, aberrant protein expression, intracellular antigen release following cell death or inflammation, and abnormal post-translational modifications [84]. The presence of anti-ALK antibodies has been reported in anaplastic large cell lymphoma (ALCL) at different time points, suggesting a possible prognostic role [85]. Additionally, both cytotoxic T-cell and CD4 T-helper responses against ALK have been identified in patients with ALK-positive tumors, not only at diagnosis but also during remission, indicating a sustained immune response [85]. In this context, Parisi et al. explored the prevalence and clinical implications of circulating anti-ALK autoantibodies (a-abs) in patients with ALK-positive NSCLC. The study analyzed plasma samples from 60 patients who had progressed on ALK tyrosine kinase inhibitors (TKIs) to assess the immunogenic potential of ALK fusion proteins. Using a semiquantitative immunocytochemical approach, anti-ALK a-abs were detected in 9 % of patients. Although their presence did not significantly affect OS or PFS, an association with a lower incidence of brain metastases was observed [86]. These findings suggest a potential role for antibody-based LB in evaluating immune responses against oncogenic drivers in ALK-positive NSCLC. Further studies are required to determine the prognostic and predictive value of anti-ALK autoantibodies and their potential role in disease monitoring and treatment response assessment. Understanding ALK immunogenicity may provide insights into tumor-host interactions and contribute to the development of novel therapeutic strategies.

## 3 Discussion and future perspectives

**Fig. 2:**
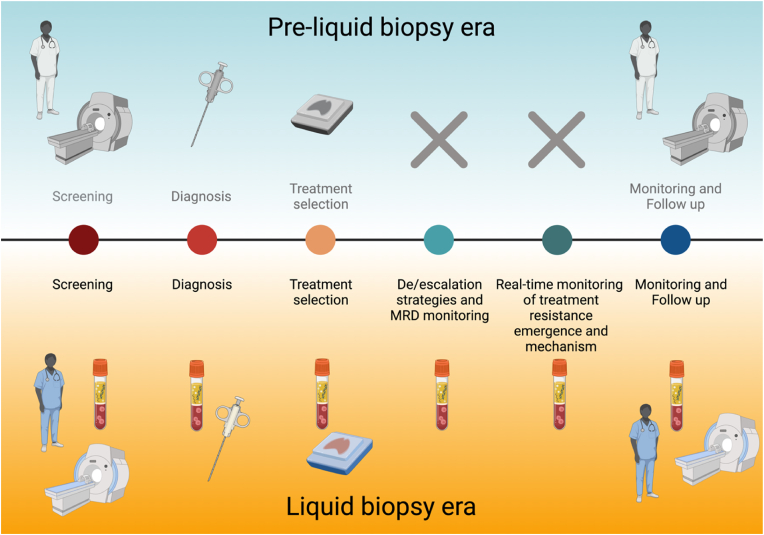
How liquid biopsy is revolutionizing the management of cancer patients Abbreviations: MRD, Minimal Residual Disease (Created in BioRender. https://BioRender.com/lxjfn00).

In the last decade, the advent of immunotherapy, targeted therapies, and antibody-drug conjugates (ADCs), have transformed cancer treatment landscape significantly improving patient outcomes [87]. In this era of precision medicine, LB is emerging as a crucial tool for screening, diagnosis, monitoring, offering a minimally invasive approach to guide treatment decisions and follow up (Fig. 2). By enabling the selection of patients most likely to benefit from specific therapies, LB not only improves clinical outcomes but also reduces the risk of unnecessary toxicities. To achieve this, continued research into novel biomarkers is essential, both at the tissue level and, more importantly, through LB, a faster, safer, and less invasive technique. The increasing integration of LB into clinical practice, supported by rapid advancements in this field, underscores its potential to refine oncology management. However, several challenges and opportunities remain in its implementation and optimization. One of the most promising applications of LB is the real-time tracking of ctDNA dynamics in patients undergoing targeted therapy, immunotherapy, or chemotherapy [88]. Studies have demonstrated that ctDNA clearance is strongly associated with improved clinical outcomes in patients treated with tyrosine kinase inhibitors (TKIs) and ICIs [89,90]. However, the critical question remains about patients who exhibit a decline in ctDNA levels but do not achieve complete clearance. These cases highlight the need for more precise response criteria and the integration of additional biomarkers to enhance patient stratification and optimize therapeutic decisions. In this context, standardized guidelines, such as the Liquid Biopsy Response Evaluation Criteria in Solid Tumors (LB-RECIST) [13], will be instrumental in defining response parameters and ensuring consistency across studies. However, harmonization of analytical methods and prospective clinical trials assessing the clinical utility of liquid biomarkers remain fundamental to advancing the field [18,91]. Beyond ctDNA, other analytes, such as EVs, CTCs, and miRNAs, are emerging as promising biomarkers for cancer monitoring. Although highly informative, evidence suggests that a multiomic approach—integrating different biomarkers with radiological imaging and clinical data—will be essential for enhancing the sensitivity and accuracy of LB [81,92]. The integration of multiple biomarkers will provide a more comprehensive understanding of tumor evolution, refine treatment strategies, and, in certain clinical scenarios, potentially replace the need for tissue biopsy. In this context, the *Journal of Liquid Biopsy (JLB)* aims to establish itself as a leading platform for researchers seeking to publish evidence in this rapidly evolving field. Since its creation, the number of original research articles published in *JLB* has increased with each volume and is expected to continue growing, attracting studies from around the world. In this mini-review, we have referenced selected articles published in *JLB* to illustrate the breadth of LB research and highlight the numerous aspects that still require further investigation. Expanding our understanding of these areas will be essential to enhancing the clinical applications of LB in the management of patients with solid tumors. Expanding our understanding of these areas will be essential to enhancing the clinical applications of LB in the management of patients with solid tumors. Ultimately, the snowball effect LB is making it an essential component of precision oncology, leading to more accurate, dynamic, and personalized cancer monitoring, and shaping the LB era.

## Ethics approval statement

Ethics approval was not required for this review.

## Declaration of competing interest

The authors declare the following financial interests/personal relationships which may be considered as potential competing interests:

Roberto Borea reports a relationship with International Society of Liquid Biopsy that includes: board membership.

Carolina Reduzzi reports a relationship with International Society of Liquid Biopsy that includes: board membership.
