# Supplementary material for: The growing field of liquid biopsy and its Snowball effect on reshaping cancer management
Source: J Liq Biopsy. 2025 Mar 27;8:100293. doi: 10.1016/j.jlb.2025.100293 (PMC12008596; doi:10.1016/j.jlb.2025.100293)
Supplement: Multimedia component 1 [file mmc1.docx]

ctDNA

(("ctDNA"[Title/Abstract] OR "circulating tumor DNA"[Title/Abstract])) AND ("tumors"[Title] OR "tumor"[Title] OR "cancer"[Title] OR "carcinoma"[Title] OR "melanoma"[Title])) AND (journal article[Publication Type] NOT review[Publication Type])

miRNA

((("miRNA"[Title/Abstract] AND "circulating" [Title/Abstract])) AND ("tumors"[Title] OR "tumor"[Title] OR "cancer"[Title] OR "carcinoma"[Title] OR "melanoma"[Title])) AND (journal article[Publication Type] NOT review[Publication Type])

CTCs

(("CTCs"[Title/Abstract] OR "circulating tumor cells"[Title/Abstract])) AND ("tumors"[Title] OR "tumor"[Title] OR "cancer"[Title] OR "carcinoma"[Title] OR "melanoma"[Title])) AND (journal article[Publication Type] NOT review[Publication Type])

EVs

((("extracellular vesicles"[Title/Abstract] AND "circulating" [Title/Abstract]) OR ("exosomes"[Title/Abstract] AND "circulating" [Title/Abstract]))) AND ("tumors"[Title] OR "tumor"[Title] OR "cancer"[Title] OR "carcinoma"[Title] OR "melanoma"[Title])) AND (journal article[Publication Type] NOT review[Publication Type])
